# Performance of the Syva Direct Fluorescent Antibody Assay for Chlamydia in a Low-Prevalence Population

**DOI:** 10.1155/S106474499300002X

**Published:** 1993

**Authors:** Mark B. Reedy, Patricia J. Sulak, William B. McCombs III, Thomas J. Kuehl

**Affiliations:** 1 Department of Obstetrics and Gynecology Scott & White Clinic and Memorial Hospital Texas A&M University College of Medicine Temple TX USA; 2 Department of Microbiology Scott & White Clinic and Memorial Hospital Texas A&M University College of Medicine Temple TX USA; 3 Department of Pathology Scott & White Clinic and Memorial Hospital Texas A&M University College of Medicine Temple TX USA; 4 Department of Medical Biochemistry and Genetics Scott & White Clinic and Memorial Hospital Texas A&M University College of Medicine Temple TX USA; 5 Department of Obstetrics and Gynecology Scott & White Clinic, 2401 S. 31st St. Temple TX 76508 USA

## Abstract

*Chlamydia trachomatis* is the most common reportable sexually transmitted disease (STD) in the United States. In the 1980s, rapid diagnostic tests for chlamydia began to replace more cumbersome tissue culture methods. Current data on rapid antigen detection assays demonstrate acceptable sensitivity, specificity, and predictive values in populations with a high prevalence of chlamydia. Few studies report the performance of these assays in a low-prevalence obstetric and gynecologic (Ob/Gyn) population, This study compares the most commonly used direct fluorescent antibody (DFA) assay (Syva Microtrak) with tissue culture (TC) in a low-prevalence population. Endocervical specimens (775) were tested from women at risk for chlamydia infection, and the prevalence was found to be 7.7%. The DFA assay demonstrated a sensitivity of 80% and a specificity of 97% compared with TC. The positive and negative predictive values were 72% and 98%, respectively. The results of this study indicate that the Syva DFA assay lacks the sensitivity and positive predictive value for routine use in Ob/Gyn populations with a lowprevalence of *C. trachomatis.*


Infectious Diseases in Obstetrics and Gynecology 1:2–6 (1993)
© 1993 Wiley-Liss, Inc.

### Performance of the Syva Direct Fluorescent Antibody Assay for Chlamydia in a Low-Prevalence Population

**Mark B. Reedy, Patricia J. Sulak, William B. McCombs III, and Thomas J. Kuehl**

*Departments of Obstetrics and Gynecology (M.B.R., P.J.S., T.J.K.), Microbiology (W.B.M.), Pathology (W.B.M., T.J.K.), and Medical Biochemistry and Genetics (T.J.K.), Scott & White Clinic and Memorial Hospital, Texas A&M University College of Medicine, Temple, TX*

### Abstract

**_Chlamydia trachomatis_ is the most common reportable sexually transmitted disease (STD) in the United States. In the 1980s, rapid diagnostic tests for chlamydia began to replace more cumbersome tissue culture methods. Current data on rapid antigen detection assays demonstrate acceptable sensitivity, specificity, and predictive values in populations with a high prevalence of chlamydia. Few studies report the performance of these assays in a low-prevalence obstetric and gynecologic (Ob/Gyn) population. This study compares the most commonly used direct fluorescent antibody (DFA) assay (Syva Microtrak) with tissue culture (TC) in a low-prevalence population. Endocervical specimens (775) were tested from women at risk for chlamydia infection, and the prevalence was found to be 7.7%. The DFA assay demonstrated a sensitivity of 80% and a specificity of 97% compared with TC. The positive and negative predictive values were 72% and 98%, respectively. The results of this study indicate that the Syva DFA assay lacks the sensitivity and positive predictive value for routine use in Ob/Gyn populations with a low prevalence of _C. trachomatis_.**
**© 1993 Wiley-Liss, Inc.**

Key words
Rapid antigen detection assay, chlamydia trachomatis, sexually transmitted disease

*Chlamydia trachomatis* is the most common reportable sexually transmitted disease in the United States.[1,2] Chlamydia is a major public health problem in the United States, with 3–5 million new cases estimated each year[1–3] and an annual economic burden of more than $1 billion.[4] The Centers for Disease Control[1] recommends chlamydia testing at the first prenatal visit and in the third trimester for high-risk patients. Clinical manifestations of the disease affect men, women, and children. Unfortunately, many genital chlamydia infections are asymptomatic in women and can result in pelvic inflammatory disease, ectopic pregnancy, and infertility.[3] The gold standard for detecting chlamydia is tissue culture (TC).[2,3,5]

Tissue culture is time consuming and expensive, and requires technical expertise. In the 1980s, rapid diagnostic tests for detecting chlamydia began replacing tissue culture methods. These antigen detection assays are simple to perform and offer rapid results. Current data on rapid antigen detection assays demonstrate acceptable sensitivity, specificity, and positive predictive value for routine use in high-prevalence populations. However, many of these assays are targeted at the private practitioner or clinic with a low prevalence of chlamydia infection. Schachter[6] has stated that nonculture methods for chlamydia antigen detection are best suited for high-prevalence populations or high-risk patients and that extrapolation of these test results to low-

**Presented at the April 27, 1992 Annual Meeting of ACOG at Las Vegas, Nevada.**
**Address correspondence/reprint requests to Dr. Mark B. Reedy, Department of Obstetrics and Gynecology, Scott & White Clinic, 2401 S. 31st St., Temple, TX 76508.**

Received May 18, 1992
Clinical Study
Accepted December 30, 1992

prevalence populations must be made with caution. Recently, the American College of Obstetrics and Gynecology Update (Precis IV) stated that the use of rapid antigen detection assays in a low-prevalence population has not been thoroughly investigated.[7] This paper describes a prospective randomized study comparing the Syva Microtrak direct fluorescent antibody (DFA) test against tissue culture (TC) in a low prevalence population.

### MATERIALS AND METHODS

### Study Population

All specimens were collected at Scott & White Memorial Hospital, Temple, Texas, from women at risk for chlamydia infection. Risk factors were: multiple sex partners, new sex partners, sexually active adolescents, pregnancy, history of other sexually transmitted disease, or signs of cervicitis. Samples were not collected from patients who received antibiotic therapy within 2 weeks of sampling. Previous studies at this hospital demonstrated the prevalence of chlamydia in this female population to be 8%.

### Collection of Specimens

All specimens were collected from the endocervix per manufacturer's instructions. Dacron swabs (Medical Wire Equipment Co., Corsham, Wilts, U.K.) were used for TC. DFA Dacron swabs were supplied in the Microtrak DFA kit. Collection order was randomized by the last digit of the medical record number (MRN). If the MRN ended in an odd number, the TC swab was collected first, followed by the DFA swab. If the MRN was even, the order was reversed. If a gonorrhea specimen or a pap smear was requested, they were collected prior to the study swabs. Swabs were placed in their respective transport containers and were tested within 24 hours of collection simultaneously by the Syva DFA and TC isolation. Specimens were stored at 4°C until tested.

### Laboratory Methods

#### *Cell Culture Methods*

Chlamydia cell culture swabs were used to inoculate McCoy cell monolayers followed by centrifugation for 1 hour at 3,000*g*. One milliliter of Chlamydia Isolator Medium with cycloheximide (Bartels Immunodiagnostics, Inc., Bellevue, WA) was added to each culture, which was then incubated at 35–37°C for 48–72 hours. A blind second passage was not performed. Cultures were stained with a *C. trachomatis*-specific fluorescent monoclonal antibody (Chlamydia Culture Confirmation Kit, Syva Co., Palo Alto, CA). Positive results were determined by identifying fluorescent-stained inclusion bodies. Results were entered in the main laboratory computer and retrieved after all testing was completed.

#### *DFA*

Slides were processed and stained according to manufacturer's instructions (Microtrak, Syva Co.). Slides were screened with a Zeiss fluorescent microscope at a ×400 magnification and confirmed at ×1,000. Slides were considered positive if ten or more elemental bodies were identified. All slides were processed and read by one of four American Society of Clinical Pathologists (ASCP) Certified Medical Technologists experienced with the Syva DFA system. All technologists are regularly tested against the CAPS (Clinical Association of Pathologists) survey slides. The DFA results were interpreted and recorded before TC results were obtained. DFA specimens were considered inadequate if less than five endocervical cells were present on a smear.

### Statistical Methods

Sensitivity, specificity, positive predictive value (PPV), and negative predictive value (NPV) were calculated for all assays. Statistical significance was determined by chi square analysis.

### RESULTS

A total of 775 endocervical specimens was tested by DFA and TC. Less than 3% of the original specimens were considered inadequate. These specimens were excluded from our final calculations. The prevalence of chlamydia based on TC results was 7.7%.

The DFA demonstrated a sensitivity of 80% and a specificity of 97%. The positive and negative predictive values were 72% and 98%, respectively (Fig. 1). There were 19 false positives and 12 false negatives.

### DISCUSSION

Many rapid diagnostic tests are available for chlamydia detection. Tissue culture is still consid-

**Results of DFA vs TC**

**TC**

| DFA | | TC + | TC - |
|---|---|---|---|
| DFA | + | 48 [a] | 19 [b] |
| | - | 12 [c] | 696 [d] |

sensitivity (a/a + c) = 80%
specificity (d/d + b) = 97%
PPV (a/a + b) = 72%
NPV (d/c + d) = 98%

Fig. 1. The results of the Syva DFA for chlamydia detection versus tissue culture isolation.

ered the gold standard by which all other assays are judged. To date, no single rapid diagnostic test for chlamydia has proved ideal for routine screening, especially in a low-prevalence (<9%) population.[7–10]

The most commonly used and studied DFA assay is the Syva Microtrak *C. trachomatis* Direct Specimen Test. Therefore, we elected to study this test in our low-prevalence population. Despite published reports[8,11,12] that used less than ten elemental bodies per slide to signify a positive DFA test, we elected to follow the manufacturer's instructions and use ten or more elemental bodies per slide for a positive test, since the majority of tests would be conducted in routine clinical laboratories and not under research protocols. Studies have shown that sensitivity can be increased by lowering the number of elemental bodies required for a positive test, but this is at the expense of lowering the specificity and positive predictive value.[9]

The DFA demonstrated a sensitivity of 80%, specificity of 97%, and PPV of 72%. This is similar to previously published reports (Table 1).[9,13–18] Stamm[10] summarized 15 Syva DFA studies in high- and intermediate-prevalence populations. In high-prevalence populations with a chlamydia prevalence of 15–26%, the cumulative sensitivity was 90% and specificity was 95%. The PPV was 90%. In populations with a prevalence of 9–11%, the sensitivity and PPV decreased to 77% and 79%, respectively.

TABLE 1. DFA (Microtrak) studies compared to TC on endocervical swabs in low-prevalence populations (< 9%)

| Author | No. of patients | Prevalence | Sensitivity | PPV |
|---|---|---|---|---|
| Gann et al.[8 a] | 268 | 7 | 53 | 69 |
| Lefebvre et al.[13 a] | 715 | 5 | 76 | 76 |
| Phillips et al.[14 a] | 527 | 4 | 70 | 62 |
| Forbes et al.[15] | 642 | 7 | 60 | 74 |
| Godfrey et al.[16] | 332 | 7 | 75 | 95 |
| Graber et al.[17] | 187 | 8 | 100 | 65 |
| Uyeda et al.[18 a] | 401 | 7 | 96 | 93 |

[a]Used less than ten elemental bodies for a positive test.

**The effect of a 3, 12, 24% prevalence on predictive values of an assay with an 80% sensitivity and a 97% specificity.**

**A. 3% prevalence (n = 1000)**

| DFA | | TC + | TC - | |
|---|---|---|---|---|
| DFA | + | 24 | 29 | PPV = 45% |
| | - | 6 | 941 | NPV = 99% |

**B. 12% prevalence (n = 1000)**

| DFA | | TC + | TC - | |
|---|---|---|---|---|
| DFA | + | 98 | 26 | PPV = 78% |
| | - | 24 | 854 | NPV = 97% |

**C. 24% prevalence (n = 1000)**

| DFA | | TC + | TC - | |
|---|---|---|---|---|
| DFA | + | 192 | 23 | PPV = 89% |
| | - | 48 | 737 | NPV = 94% |

Fig. 2. How prevalence affects positive predictive value.

Predictive values are influenced by prevalence rates in a population.[2,6,11] Grimes[19] stated that when screening for disease in a low-prevalence population, even with a high sensitivity and specificity, the PPV would be low. Figure 2 illustrates how prevalence affects PPV.

When interpreting these data, one must be aware that TC is not the perfect gold standard. The sensitivity of isolating chlamydia from a single swab is estimated to be 70–80%.[2,5,6,9] The use of immunofluorescent staining of TC increases the sensitivity similar to that of a blind second passage.[10] Even with a blind second passage of fluorescent antibody staining, TC will not identify all infected patients.

There were 19 false positives; six of these also tested positive by another antigen detection assay (TestPack Chlamydia Abbott Labs, Chicago, IL). Several authors[8,11,12,20–22] have used a second antigen detection assay to demonstrate TC false negatives. If both antigen detection methods were positive, they would consider the specimen as a true positive and the TC as a false negative. If these six specimens are considered true positives, the sensitivity of the Syva DFA increases from 80% to 82% and the specificity from 97% to 98%. The PPV increases from 72% to 80%. The original and adjusted statistics are not significantly different ($P > 0.05$).

If TC has a sensitivity of 80% and the Syva DFA assay detects 80% of TC positives, then only 65% of infected patients are identified with the DFA assay. This appears to be an unsatisfactory detection record for a treatable infection that affects a broad spectrum of society.

Also of concern is the low PPV. Between 20% and 30% of the Syva DFA positives will be false positives in this population. A false positive result on an STD test may have grave social implications. Thus, some authors advocate TC confirmation of positive DFA results when used in a low-prevalence population.[23] Antigen detection assays have demonstrated unequivocal false positive results in patients evaluated for sexual abuse.[24] The Centers for Disease Control (CDC) recommends only standard tissue culture methods to identify *C. trachomatis* in the evaluation of sexual abuse.[1] Tissue culture isolation is still the most sensitive and specific diagnostic test for chlamydia.[9,10,22]

Recent data show that DFA sensitivity may be increased by the use of the cytobrush to sample the endocervix.[25,26] The cytobrush was not used in this study because the manufacturer did not recommend its use at the initiation of the study. The Syva Company still does not recommend the use of the cytobrush in pregnancy.

The results of our study indicate that the Syva DFA assay lacks the sensitivity and PPV for routine screening in Ob/Gyn populations with a low prevalence of *C. trachomatis*. With the advent of newer technologies for the detection of chlamydia (i.e., DNA probes and polymerase chain reaction), the private practitioner or clinic should critically analyze the performance of these assays in their targeted patient population before beginning widespread screening.

### REFERENCES

1. Centers for Disease Control: 1989 Sexually transmitted diseases treatment guideline. MMWR 38(S-8):1–43, 1989.
2. Sweet RL, Gibbs RS: Chlamydial infections. In: Infectious Diseases of the Female Genital Tract. 2nd ed. Baltimore: Williams & Wilkins, pp 45–74, 1990.
3. Martin DH: Chlamydial infections. Med Clin North Am 74:1367–1387, 1990.
4. Washington AE: Chlamydia: A major threat to reproductive health. *Research Highlights,* Institute for Health Policy Studies. University of California, San Francisco 2:1–3, 1984.
5. Holmes KK, Mardh P-A, Sparling PF, Wiesner PJ, Cates W, Jr, Lemon SM, Stamm WE: *Chlamydia trachomatis*. In: Holmes KK et al. (eds): Sexually Transmitted Diseases. 2nd ed. New York: McGraw-Hill, pp 917–925, 1990.
6. Schachter J: Rapid diagnosis of sexually transmitted diseases—speed has a price. Diagn Microbiol Infect Dis 4:185–189, 1986.
7. American College of Obstetrics and Gynecology: *Precis IV* ACOG, p 31, 1990.
8. Gann PH, Herrmann JE, Candib L, Hudson RW: Accuracy of *Chlamydia trachomatis* antigen detection methods in a low-prevalence population in a primary care setting. J Clin Microbiol 28:1580–1585, 1990.
9. Barnes RC: Laboratory diagnosis of human chlamydial infections. Clin Microbiol Rev 2:119–136, 1989.
10. Stamm WE: Diagnosis of *Chlamydia trachomatis* genitourinary infections. Ann Intern Med 108:710–717, 1988.
11. Schwebke JR, Stamm WE, Handsfield HH: Use of sequential enzyme immunoassay and direct fluorescent antibody tests for detection of *Chlamydia trachomatis* infections in women. J Clin Microbiol 28:2473–2476, 1990.
12. Baselski VS, McNeeley SG, Ryan G, Robison M: A comparison of nonculture-dependent methods for detection of *Chlamydia trachomatis* infections in pregnant women. Obstet Gynecol 70:47–52, 1987.
13. Lefebvre J, Laperiere H, Rousseau H, Masse R: Comparison of three techniques for detection of *Chlamydia trachomatis* in endocervical specimens from asymptomatic women. J Clin Microbiol 26:726–731, 1988.
14. Phillips RS, Hanff PA, Kauffman RS, Aronson MD: Use of a direct fluorescent antibody test for detecting *Chlamydia trachomatis* cervical infection in women seeking routine gynecologic care. J Infect Dis 156:576–581, 1987.
15. Forbes BA, Bartholoma N, McMillan J, Roefaro M, Weiner L, Welych L: Evaluation of a monoclonal antibody test to detect chlamydia in cervical and urethral specimens. J Clin Microbiol 23:1136–1137, 1986.
16. Godfrey E, Winn W, Jr, Keathley JD. Performance of

Microtrak direct test for *Chlamydia trachomatis* in a prevalence study. Diagn Microbiol Infect 5:313–316, 1986.

17. Graber CD, Williamson O, Pike J, Valicenti J: Detection of *Chlamydia trachomatis* infection in endocervical specimens using direct immunofluorescence. Obstet Gynecol 66:727–730, 1985.
18. Uyeda CT, Welborn P, Ellison-Birang N, Shunk K, Tsaouse B: Rapid diagnosis of chlamydial infection with the MicroTrak direct test. J Clin Microbiol 20:948–950, 1984.
19. Grimes DA: Screening tests. What they are and what they aren't. Contemp OB/GYN 20:69–74, 1982.
20. Moncada J, Schachter J, et al: Confirmatory assay increases specificity of chlamydiazyme test for *Chlamydia trachomatis* infection of the cervix. J Clin Microbiol 28: 1770–1773, 1990.
21. Gaydos CA, Reichart CA, et al.: Evaluation of Syva enzyme immunoassay for detection of *Chlamydia trachomatis* in genital specimens. J Clin Microbiol 28:1541–1544, 1990.
22. Binn B, Williams JT, McDowell J, Brunham RT, Brunham RC: Screening for *Chlamydia trachomatis* infection in a pregnant counseling clinic. Am J Obstet Gynecol 15a: 1144–1149, 1988.
23. Martens MG: Office diagnosis of sexually transmitted diseases. Obstet Gynecol Clin North Am 16:659–677, 1989.
24. Dorian KJ, Holtzhauer F, et al.: False-positive results with the use of chlamydia tests in evaluation of suspected sexual abuse—Ohio, 1990. MMWR 39:932–935, 1991.
25. Weiland TL, Noller KL, Smith TF, Ory SJ: Comparison of Dacron-tipped applicator and cytobrush for detection of chlamydial infections. J Clin Microbiol 26:2437–2438, 1988.
26. Moncada J, Schacter J, Shipp M, Bolan G, Wilber J: Cytobrush in collection of cervical specimens for detection of *C. trachomatis*. J Clin Microbiol 27:1863–1866, 1989.
